# A Chest Pain Conundrum: Dapsone-Induced Methemoglobinemia in a Heart Transplant Patient

**DOI:** 10.7759/cureus.46690

**Published:** 2023-10-08

**Authors:** Kristina Akopyan, Konstantin Golubykh, Jared Freitas, Borna Mehrad

**Affiliations:** 1 Internal Medicine, University of Florida, Gainesville, USA; 2 Internal Medicine, University of Pittsburgh Medical Center, Harrisburg, USA; 3 Pulmonology and Critical Care, University of Florida, Gainesville, USA; 4 Pulmonary and Critical Care Medicine, University of Florida, Gainesville, USA

**Keywords:** cytochrome b5 reductase, saturation gap, methylene blue treatment, refractory hypoxemia, dapsone-induced methemoglobinemia

## Abstract

We present the case of a 39-year-old male with a past medical history of orthotopic heart transplantation who presented with chest pain and dyspnea on exertion. He was diagnosed with dapsone-induced methemoglobinemia toward the end of his hospital course, and his condition clinically improved with the discontinuation of the offending agent. This case highlights the importance of medication review and history-taking. Clinicians should be mindful of dapsone-induced methemoglobinemia, especially when encountering patients with dyspnea and a history of dapsone intake.

## Introduction

Dapsone-induced methemoglobinemia is a rare disorder, but it can be potentially fatal if left untreated. Dapsone is a sulfonamide derivative, which is used for the treatment of malaria, leprosy, dermatitis herpetiformis, and prophylaxis of Pneumocystis pneumonia [1]. Some of the known adverse effects of dapsone include agranulocytosis, nephritis, Stevens-Johnson syndrome, and methemoglobinemia [2]. Methemoglobin is a form of hemoglobin in which the heme iron is oxidized from the ferrous to the ferric state. Hypoxemia results due to the low oxygen-binding capacity of the ferric state [3]. Methemoglobin is maintained by two different mechanisms: the cytochrome b5 reductase pathway, and the nicotinamide adenine dinucleotide phosphate (NADPH)-dependent methemoglobin reductase, which requires a cofactor like methylene blue or riboflavin to be activated [4]. We discuss a case of a patient whose complex condition masked a straightforward solution.

## Case presentation

A 39-year-old male with a past medical history significant for orthotopic heart transplantation secondary to non-ischemic cardiomyopathy complicated by antibody-mediated rejection, who was on rituximab every 12 weeks, with non-sustained ventricular tachycardia status post automatic implantable cardioverter-defibrillator (AICD) presented to the emergency department for chest pain, which was associated with shortness of breath, for the last two weeks. The chest pain was described as severe and intermittent, radiating to the left shoulder and arm and associated with increased dyspnea on exertion. He denied having palpitations, orthopnea, or lower extremity swelling. He also denied having any fevers, chills, sick contacts, or changes to medications including immunosuppressives.

On physical examination, the patient was saturating 93% on 2 L/minute of nasal cannula oxygen; he had been initially saturating 89% on room air, had a blood pressure of 144/75 mmHg, respiratory rate of 14 per minute, pulse of 95 beats per minute, and temperature of 98.5 °F. He was in no acute distress and was tachycardic with regular rhythm and normal pulses. He had no murmurs and no jugular venous distention. His lungs were clear to auscultation bilaterally. The abdomen was soft, non-distended, non-tender, with positive bowel sounds. His skin was warm and dry, and he had no edema in the lower extremities. Laboratory studies were significant for normocytic anemia, elevation in brain natriuretic peptide (BNP), and elevation in troponin I high sensitivity (Table 1).

**Table 1:** Laboratory findings on admission MCV: mean corpuscular volume; BNP: brain natriuretic peptide

Laboratory parameters	Laboratory findings	Reference range
Hemoglobin	8.3 g/dL	13.0-16.5 g/dL
Hematocrit	25.2%	39.0-49.0%
MCV	85.1 FL	78.0-100.0 FL
BNP	431 pg/mL	<100 pg/mL
Troponin I high sensitivity	43 pg/mL	<20 pg/mL

**Figure 1:**
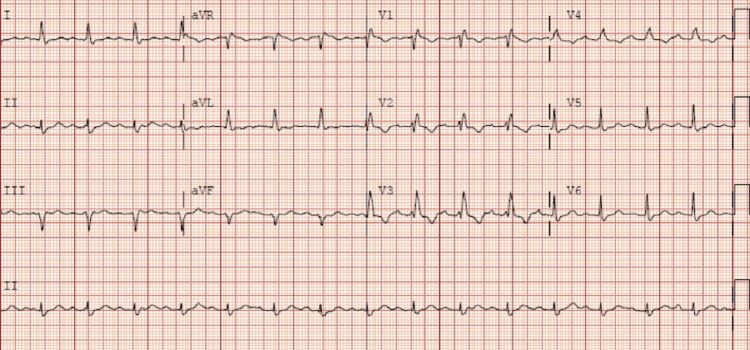
EKG on admission showing first-degree AV block EKG: electrocardiogram; AV: atrioventricular

EKG showed a first-degree AV block (Figure 1).

AICD was examined and no arrhythmias were noted. Transthoracic echocardiogram showed normal left ventricular function with an ejection fraction (EF) of 55-60% and estimated right ventricular systolic pressure (RVSP) of 40-45 mmHg.

**Figure 2:**
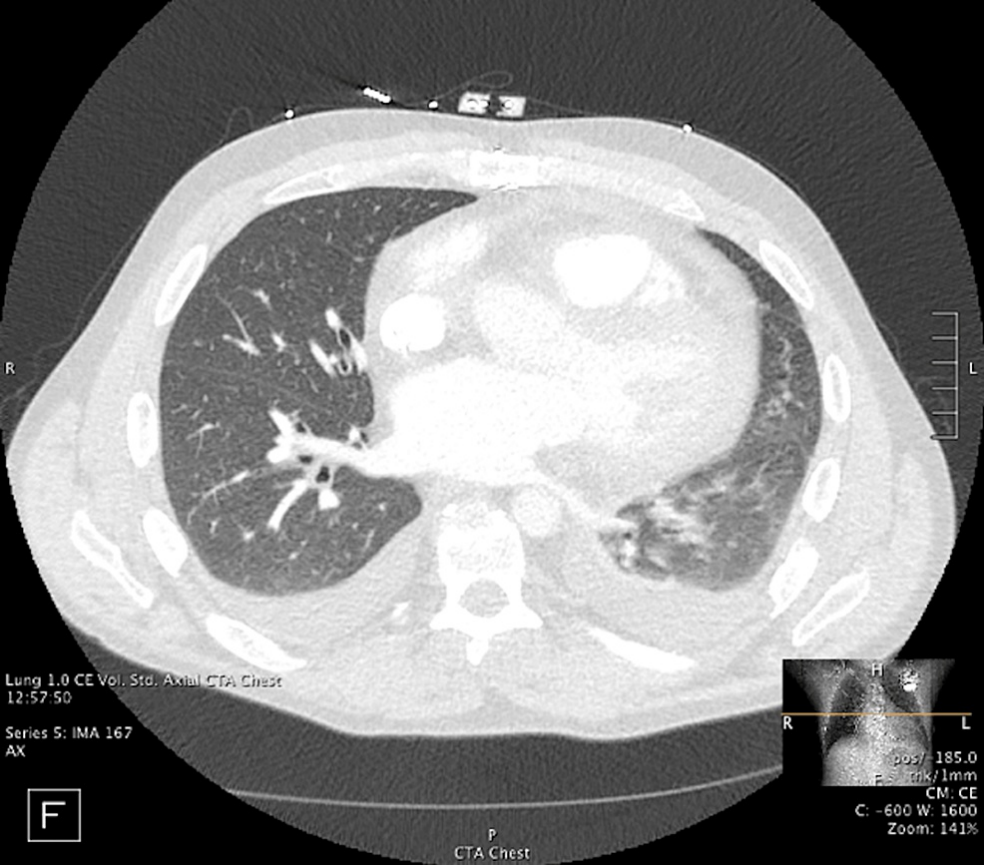
CTA showing evidence of mild pulmonary edema and bilateral pleural effusions CTA: computed tomography angiography

The patient was admitted to the heart transplant service. He received a blood transfusion due to suspicion that his symptoms were caused by anemia. Despite the blood transfusion, his dyspnea continued to worsen along with an increase in oxygen requirement to 3 L/minute of nasal cannula oxygen with SpO_2_ of around 90%. CT angiography (CTA) showed no evidence of pulmonary embolism. However, there was evidence of mild pulmonary edema and small bilateral pleural effusions (Figure 2).

The patient was given IV bumetanide 2 mg twice daily. He underwent a left heart catheterization (LHC) and right heart catheterization (RHC). The RHC was significant for mildly elevated filling pressures with normal wedge pressure and normal cardiac output (Table 2).

**Table 2:** RHC Findings RHC: right heart catheterization; PCWP: pulmonary capillary wedge pressure; PAP: pulmonary artery pressure; RVP: right ventricular pressure; RAP: right atrial pressure; CO: cardiac output

RHC parameters	RHC findings	Reference range
PCWP	8 mmHg	6-12 mmHg
PAP (systolic/diastolic/mean)	23/10/17 mmHg	15-30/8-15/9-18 mmHg
RVP (systolic/diastolic)	23/8 mmHg	25/5 mmHg
RAP	8 mmHg	2-6 mmHg
CO	5.66 L/minute	4.0-8.0 L/minute

The LHC showed coronary artery vasculopathy, which was unchanged from the prior study. The diuretic therapy was then discontinued given findings were not consistent with volume overload. Hematology was consulted and they stated that the degree of anemia could not explain his dyspnea, while ruling out all other causes of anemia, such as iron deficiency anemia, vitamin b12 deficiency, and folic acid deficiency. Pulmonary medicine was then consulted due to persistent hypoxia and dyspnea. During history-taking, the patient mentioned the unchanging “number on the monitor” regardless of oxygen supplementation, referring to the SpO_2_ percentage. This observation prompted suspicion of dapsone-induced methemoglobinemia. Co-oximetry on arterial blood gas (ABG) was subsequently ordered, and the methemoglobin level returned elevated at 9.7% (Table 3).

**Table 3:** Co-oximetry on ABG ABG: arterial blood gas

ABG parameters	ABG findings	Reference range
pH	7.45	7.35-7.45
pCO_2_	43.9 mmHg	35.0-45.0 mmHg
pO_2_	67.3 mmHg	90.0-105.0 mmHg
O_2 _saturation	92.1%	95.0-99.0%
Carboxyhemoglobin	0.0%	0.0-2.0%
Methemoglobin	9.7%	0.0-0.9%

Dapsone was then immediately discontinued, and the patient’s dyspnea with chest pain subsequently resolved. He no longer required supplemental oxygen, and he was discharged home in a stable condition.

## Discussion

The diagnosis and treatment of methemoglobinemia can be challenging. Patients might present with a variety of different symptoms including dyspnea, nausea, cyanosis, chest pain, stupor, and loss of consciousness in the most severe cases [5]. The diagnosis is often delayed as methemoglobin levels are not always readily available and pulse oximetry can be misleading [6]. Methemoglobinemia is diagnosed when there is a discrepancy between the SpO_2_ and SaO_2_ that is refractory to oxygen therapy known as the “saturation gap”, the presence of chocolate-colored blood, and signs of cyanosis [5]. The saturation gap occurs because of the limitations of pulse oximetry. Pulse oximetry can only measure oxyhemoglobin and reduced hemoglobin.

Methemoglobinemia causes the oxygen saturation to fall and plateau at 85% [6]. For example, our patient’s SaO_2_ was 92.1%, and the SpO_2_ at that time was 89%. This difference in the SaO_2_ and SpO_2_ was suggestive of alternative forms of hemoglobin, such as methemoglobin. The patient’s hemoglobin level of 8.3 g/dL could not explain his chest pain and dyspnea on exertion. However, some of the hemoglobin was unavailable for gas exchange, which may have contributed to his symptoms and refractory hypoxemia. The initial treatment for dapsone-induced methemoglobinemia should be the immediate discontinuation of the offending agent. Methylene blue is recommended for asymptomatic patients when the percentage of methemoglobinemia is 30 or greater and for symptomatic patients when the percentage of methemoglobinemia is 20 or greater [5]. There is a risk of hemolytic anemia and worsening methemoglobinemia in patients with glucose-6-phosphate dehydrogenase (G6PD) deficiency [7]. Methylene blue acts as an electron donor by NADPH-methemoglobin and reduces methemoglobinemia. Ascorbic acid and charcoal are other alternatives to methylene blue [8].

## Conclusions

This case underscores the importance of a comprehensive medication review and detailed history-taking in cases of complex presentations, particularly in post-transplant patients. In our patient, the chief complaint of chest pain led to an extensive workup, indicating the propensity among physicians to opt for intricate evaluations. However, the root of the problem was a relatively simple and correctable medication side effect. Dapsone-induced methemoglobinemia, while a rare occurrence, can be potentially fatal if overlooked. Clinicians should be knowledgeable about its presentation and maintain a high index of suspicion, especially when managing dyspnea in patients with a known history of dapsone intake. This case highlights the importance of obtaining co-oximetry on ABG in patients presenting with dyspnea and a history of dapsone intake to rule out dapsone-induced methemoglobinemia. In complex cases, it is crucial to remember that sometimes the solution lies not in the intricate depths but on the surface.

## References

[1] Trillo RA Jr, Aukburg S (1992). Dapsone-induced methemoglobinemia and pulse oximetry. Anesthesiology.

[2] Keerty D, Eaton K, Haynes E (2020). Dapsone-induced hypoxia. Cureus.

[3] Khan Suheb M, Naaz F, Anderson TK, McClanahan A (2022). A case of cyanosis with saturation gap: dapsone-induced methemoglobinemia. Cureus.

[4] Mahmood N, Khan MU, Haq IU, Jelani FA, Tariq A (2019). A case of dapsone-induced methemoglobinemia. J Pharm Policy Pract.

[5] Cefalu JN, Joshi TV, Spalitta MJ (2020). Methemoglobinemia in the operating room and intensive care unit: early recognition, pathophysiology, and management. Adv Ther.

[6] Burke P, Jahangir K, Kolber MR (2013). Dapsone-induced methemoglobinemia: case of the blue lady. Can Fam Physician.

[7] Lewis JS, Jacobs ZG (2020). Subtle case of dapsone-induced methaemoglobinaemia. BMJ Case Rep.

[8] El Hamzaoui H, Chajai I, El Ouazzani MC, Benhalima A, El Arfaoui M, Alilou M (2022). Acute dapsone poisoning with methemoglobinemia: a case report. Pan Afr Med J.

